# Psychobiological factors of resilience and depression in late life

**DOI:** 10.1038/s41398-019-0424-7

**Published:** 2019-02-14

**Authors:** Kelsey T. Laird, Beatrix Krause, Cynthia Funes, Helen Lavretsky

**Affiliations:** Department of Psychiatry, Semel Institute for Neuroscience and Human Behavior at UCLA, Los Angeles, CA USA

## Abstract

In contrast to traditional perspectives of resilience as a stable, trait-like characteristic, resilience is now recognized as a multidimentional, dynamic capacity influenced by life-long interactions between internal and environmental resources. We review psychosocial and neurobiological factors associated with resilience to late-life depression (LLD). Recent research has identified both psychosocial characteristics associated with elevated LLD risk (e.g., insecure attachment, neuroticism) and psychosocial processes that may be useful intervention targets (e.g., self-efficacy, sense of purpose, coping behaviors, social support). Psychobiological factors include a variety of endocrine, genetic, inflammatory, metabolic, neural, and cardiovascular processes that bidirectionally interact to affect risk for LLD onset and course of illness. Several resilience-enhancing intervention modalities show promise for the prevention and treatment of LLD, including cognitive/psychological or mind–body (positive psychology; psychotherapy; heart rate variability biofeedback; meditation), movement-based (aerobic exercise; yoga; tai chi), and biological approaches (pharmacotherapy, electroconvulsive therapy). Additional research is needed to further elucidate psychosocial and biological factors that affect risk and course of LLD. In addition, research to identify psychobiological factors predicting differential treatment response to various interventions will be essential to the development of more individualized and effective approaches to the prevention and treatment of LLD.

## Depression vs. resilience in late life

Late-life depression (LLD) is a common and debilitating condition, with less frequent remission and more frequent recurrence following first-line antidepressant treatment compared to depression experienced earlier in life^1–7^. Factors contributing to LLD are multifaceted, including biological (e.g., genetic), psychological (e.g., personality), and social influences (e.g., social support). With the world population rapidly aging, it is increasingly important to identify factors that increase resilience to the development and maintenance of LLD.

Psychological resilience has been broadly defined as “the capacity to maintain, or regain, psychological well-being in the face of challenge”^8^. Resilience is a complex construct that can be conceptualized as an *attribute* (a trait) that is possessed to varying degrees by different individuals, a dynamic *process* (a state) with bidirectional relations to developmental and environmental factors, and as an *outcome* in the face of stress and adversity^9^. Depending on the theoretical perspective, population, and risk factor in question, resilient outcomes may be operationalized as either the presence of a positive outcome (e.g., life satisfaction) or the absence of a negative one (e.g., lack of psychopathology)^10^. We conceptualize psychological resilience as a multidimensional, dynamic capacity influenced by the interaction of internal factors (e.g., cognitive capacity, personality, physical health) and external resources (e.g., social status, financial stability)^11^. In the context of major depressive disorder (MDD), psychological resilience refers to the net effects of a variety of psychosocial and biological variables that decrease risk of onset or relapse, decrease illness severity, or increase probability or speed of recovery. The current review describes resilience and vulnerability factors related to LLD. We summarize psychosocial resilience factors that are universal across age groups as well as those unique to aging. We also present results of research investigating the neurobiological, genetic, and immunological biomarkers of resilience.

## Psychological resilience factors

**Fig. 1:**
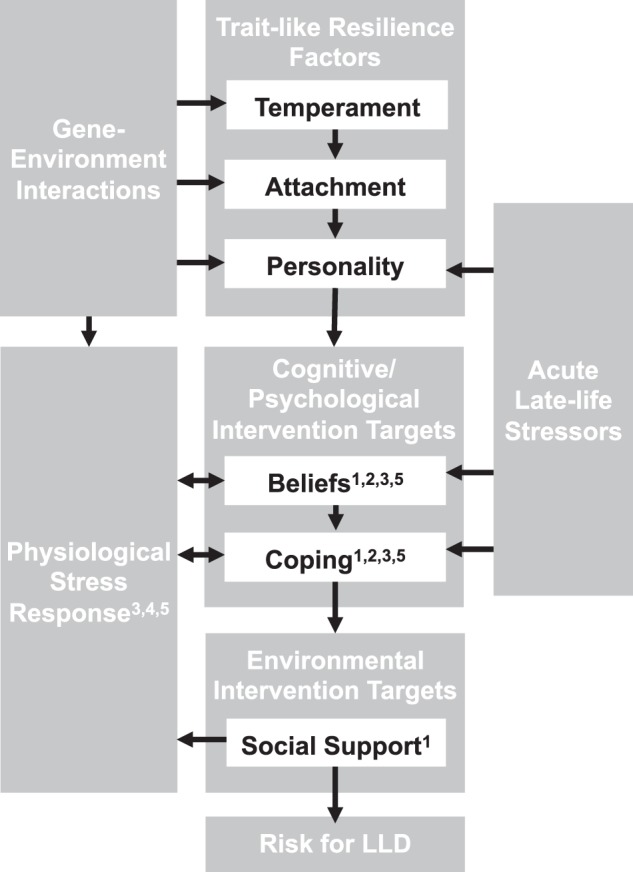
Biopsychosocial factors influencing risk for LLD onset and course. ^1^ Target of positive-psychology interventions and psychotherapy. ^2^ Target of mindfulness-based interventions. ^3^ Target of movement-based interventions. ^4^ Target of pharmacotherapy and electroconvulsive therapy. ^5^Target of heart rate variability biofeedback

Multiple psychological resilience factors reduce an individual’s risk for depression across the lifespan^12^. Enduring individual characteristics such as temperament, attachment style, and personality each prospectively predict risk for depression. In addition, multiple psychological processes are proposed to mediate this effect, including beliefs and coping behavior. Psychosocial and biological correlates of LLD are presented in Table 1. A proposed model of how biopsychosocial factors influence risk for LLD and illness course is presented in Fig. 1.

**Table 1:** Biopsychosocial correlates of late-life depression (LLD)

Psychosocial factors	Resilience correlates	LLD correlates
Temperament	Positive emotionality	Behavioral inhibition
Attachment	Secure attachment	Insecure attachment
Personality	Extroversion, conscientiousness, grit	Neuroticism
Beliefs	Self-esteem, self-efficacy, mastery, growth mindset, sense of purpose	Depression-related stigma, negative attitudes about aging
Coping	Active coping, accommodative coping, religious/spiritual practice	Passive coping
Social factors	Social support, formal volunteering	Trauma, chronic stress, more social role "absences", loneliness
Lifestyle factors	Physical exercise, healthy diet	Sedentary lifestyle, nutritional deficiencies, substance abuse

Biological factors	Resilience correlates	LLD correlates
Genetics	Val/Val allele, higher expression of mineralocorticoid receptors	Val/Met allele, APOE-4e, SLC6A4, female sex
Neurophysiological	Higher methylation of BDNF, higher neuropeptide Y, efficient monoamine transmission	Neurodegeneration, white matter hyperintensities/vascular deficiencies, shortened telomeres, lower heart rate variability, hippocampal atrophy
Steroid hormones	Higher dehydroepiandrosterone (DHEA), moderate availability of estrogens	Lower DHEA, low or very high availability of estrogens

### Temperament

Temperament is a basic inherited style, the structure of which has been inferred largely from genetic studies. Meta-analytic data indicate that within the temperament dimensions, harm avoidance (i.e., “behavioral inhibition”) is associated with greater MDD risk and decreased treatment responsivity^13^. Research on individuals with age-related illness suggests that harm avoidance is similarly predictive of MDD in late life^14,15^. In contrast, meta-analytic data indicate that positive emotionality (e.g., positive affect, extraversion, and behavioral activation) decreases risk for depression^16^. Related longitudinal research suggests that the capacity to experience positive emotions such as gratitude, interest, and love is one mechanism by which resilient individuals are buffered against risk for depression following trauma^17^.

### Attachment

Because early life attachment is thought to shape subsequent relationships, attachment theory^18–20^ offers an important framework for understanding the etiology and development of depression across the lifespan^21^. Insecure attachment is a risk factor for depression onset^22^, illness severity, and relapse^23^. Research suggests that increased emotional awareness^24^ and coping self-efficacy^25,26^ may be two mechanisms by which secure attachment decreases stress reactivity. Longitudinal studies^27^ as well as studies of children raised in orphanages^28^ indicate that disorganized attachment during infancy predicts greater amygdala volume—a hypothesized biomarker of difficulty with emotion regulation^28^. Although attachment style remains moderately stable throughout life, stressful life events have potential to decrease secure attachment^29^, while high relationship satisfaction and emotional openness may increase secure attachment^29^. Research indicates that insecure attachment continues to increase risk for depression in late life^30,31^.

### Personality

Multiple personality factors increase risk for MDD. A recent meta-analysis controlling for baseline depressive symptoms found that low extraversion, high neuroticism, and low conscientiousness predicted depressive symptoms 5 years later^32^. Research on stroke survivors^14^, individuals with Parkinson’s Disease^33^, and nonclinical older adult samples^34^ has found similar associations, suggesting that these effects are also observed in late life. Another personality characteristic associated with resilience is grit, defined as “perseverance and passion for long-term goals” in the face of setbacks^35^. A recent study of 337 adults with LLD found that grit was associated with decreased severity of depression, apathy, and anxiety^36^.

### Beliefs and coping behavior

Cognitive behavioral theory posits that an individual’s beliefs about themselves, others, and the environment influence coping behavior and subsequent psychosocial adjustment^37,38^. For example, two recent meta-analyses found that low self-esteem (i.e., a negative evaluation of one’s self-worth) prospectively increased risk for depression^39,40^. Similarly, an individual’s self-efficacy for coping with a given stressor impacts the coping strategy they select and how long they persist in their efforts^41^. Coping can be broadly divided into two domains: active (efforts to directly “solve” the source of stress) and accommodative (efforts to accept or adapt to the source of stress)^42^. Research indicates that the ability to flexibly apply active vs. accommodative strategies—i.e., using active approaches for controllable stressors and accommodative approaches for uncontrollable stressors—results in more favorable mental health outcomes^43–49^. A recent study of 337 adults with LLD found that both active coping self-efficacy and accommodative coping self-efficacy were associated with decreased depressive symptoms, apathy, and anxiety^36^. In addition, greater baseline accommodative coping self-efficacy predicted antidepressant treatment responsivity^50^. In general, individuals with high self-efficacy for managing stress through adaptive approaches such as physical exercise, social support, and self-care are more likely to engage in these strategies and less likely to develop prolonged symptoms of depression. In contrast, low coping self-efficacy is associated with passive coping^51^, avoidance, lower treatment adherence^52,53^, substance use^54^, and other maladaptive coping strategies^55^ that may serve to increase risk or course of depression.

An internal locus of control (i.e., “mastery”)^56^ is the general belief in one’s ability to influence outcomes^57^. Because this belief increases self-efficacy for coping with a range of stressors, mastery can be conceptualized as another resilience factor. Similarly, evidence suggests that a “growth mindset”—the belief that one’s abilities can be enhanced through effort—increases resilience by increasing grit^58^. Finally, a strong sense of meaning/purpose in life increases resilience to depression across the lifespan^59^. A study of 1475 older Australian adults found that higher sense of purpose was associated with less disability, higher neurocognitive performance, and decreased depressive symptoms, and predicted increased survival in late life^59^. By contrast, depressed older adults with symptoms of apathy generally have poorer clinical outcomes^60^, quality of life (QOL)^61,62^, treatment response^63,64^, cognitive impairment^61^, and disability^62^, possibly resulting from decreased engagement in social- and health-related behaviors^62^. Other studies have investigated the effect of meaning/purpose on risk for depression among those at high risk due to exposure to acute or chronic stress. A longitudinal study of bereaved adults showed that two construals of meaning—making sense of the loss and finding benefit in the experience—both independently predicted decreased depressive symptoms^65^. Other studies conducted with cancer survivors^66^ and individuals with terminal illness^67^ also report associations between meaning and decreased depressive symptoms. The results of one study suggest that increases in perceived meaning and benefit finding may be one way by which cognitive behavioral therapy helps prevent depression among cancer patients^68^.

A final category of beliefs that increases risk for poor outcomes in depression is mental illness stigma^69^. A recent meta-analysis found strong associations between internalized mental illness stigma and poorer psychological resources (hope, self-esteem, empowerment), lower treatment adherence, and greater mental illness symptom severity^70^. In a study of adults with LLD, higher baseline depression-related stigma predicted worse treatment response, after controlling for baseline depression severity^70,71^. However, results of a longitudinal study of adults with LLD suggest that mastery may moderate this effect^72^. In that sample, anticipated stigma only predicted increased depressive symptoms among those with low mastery^72^. Mastery may counteract the negative impact of anticipated stigma on mental health by increasing older adults’ confidence in their ability to cope with stressors such as interpersonal rejection.

### Religion and spirituality

Religion and spirituality have been shown to prospectively reduce risk for depression^73^. Whether such effects are attributable to religious beliefs, behavior, or social support remains a matter of debate. One investigation of this question involved a study of over 1000 adult Detroit residents^74^. In that study, religious attendance (e.g., church, temple, synagogue) was associated with greater psychological well-being and less emotional distress; frequency of prayer was associated with lower well-being and more distress; and belief in eternal life was associated with greater well-being but unrelated to distress. Religious attendance was associated with lower distress even after controlling for sociodemographic variables (e.g., age, sex, education), stressors (e.g., health problems, financial problems), social resources (family contact, support, and negative interaction), and psychological resources (self-esteem and personal mastery). By contrast, a study of almost 3000 older Taiwanese adults found that religious attendance no longer predicted decreased depressive symptoms after controlling for health behaviors, social networks, and supports^75^. Consistent with the results of the Detroit study, religious beliefs and depressive symptoms were unrelated. Finally, a study of almost 8000 US older adults found that frequent attendance of religious services predicted decreased depression onset and frequent private prayer predicted increased depression remission 2 years later. Results of these studies indicate that religious coping behaviors may be more strongly protective against depression than religious beliefs.

## Psychological factors specific to late life

The types of stressors encountered by older adults are qualitatively different than those faced by younger age groups. Late-life stressors include voluntary or forced retirement, chronic illness, cognitive decline, caregiving, financial stress, loss of independence, and bereavement. If these types of stressors are less controllable than those encountered by younger age groups, this could make accommodative coping especially essential in geriatric populations^76^. Indeed, older adults appear to engage in more accommodative coping^77^ and less instrumental action coping^76^ compared to younger adults. Wrosch and colleagues propose a developmental theory in which age-adapted selection of coping strategies relates to optimal well-being^49^. Consistent with this theory, active coping (i.e., “persistence”) was significantly associated with well-being in young adulthood and midlife, but not late life^49^. Among older adults, accommodative coping was more strongly associated with psychological well-being than was persistence. Another study of LLD found that accommodative coping self-efficacy was uniquely predictive of subsequent remission^50^.

### Attitudes and stereotypes

One category of beliefs especially relevant to resilience in late life includes attitudes about aging itself. Studies in which negative stereotypes about aging are experimentally activated have found that both implicit/subconscious and explicit/conscious stereotypes negatively impact performance in older people attempting physical and cognitive tasks^78,79^. Other studies employing a cross-cultural approach have found larger age differences in cognitive performance in cultures with more negative stereotypes^80,81^.

### Social role and identity changes

Traditional perspectives have assumed that major life changes inherently stressful^82^. However, research suggests that role transitions such as retirement exert a wide range of possible mental health effects^83–85^, including increasing well-being when the change represents an escape from a chronically stressful role situation^86^. A large Canadian survey found that retiring from a low-stress job increased depressive symptoms compared to not retiring, whereas retiring from a high-stress job resulted in an effect twice as large in the opposite direction^86^.

Several studies have found that a greater number of “absences” in major social role-identities (marital, parental, and employment) is associated with poor late-life psychological adjustment^87–89^. Cumulating evidence indicates that formal volunteering may buffer against this effect by increasing social engagement, life satisfaction, self-worth, personal growth, and sense of purpose/meaning^87,90–93^. For example, a US study of almost 400 older adults found that a greater number of major role-identity absences was associated with more negative affect, less positive affect, and less purpose in life^87^. Formal volunteering was associated with positive affect, and volunteering positively moderated the relationship between role-identity absences and purpose in life^87^. Other studies have found that older adults typically experience greater increases in life satisfaction with volunteering compared to younger adults^91^ and that adoption of a volunteer role may offset the negative impact of spousal bereavement on subsequent depressive symptoms^94^.

## Social resilience factors

Systematic reviews suggest that both perceived social support and objective social network size protect against depression in the general population^95^ as well as in older adults specifically^96^. Research investigating the mechanisms by which social networks enhance psychological resilience indicates that both emotional support and tangible (“instrumental”) support are important contributors^82^. Research has also begun to distinguish between the effects of objective vs. perceived social isolation (i.e., loneliness)^97^. One study of over 1300 older Irish adults concluded that these constructs were distinct and that each independently predicted depressive symptoms^98^. An even larger US study of 20,000 adults found that loneliness was correlated with a host of other risk factors—less physical exercise, lower sleep quality, lower social engagement, and poorer physical health^99^. Each of these factors likely interact to predict susceptibility to MDD. A cross-cultural review of additional social resilience factors identified being married or cohabiting, male, and having a higher family income each as associated with reduced risk of depression in the US and Japan^100^. However, a 3-year longitudinal study of American adults aged 50–67 found that loneliness uniquely predicted depressive symptoms after controlling for demographic and psychosocial covariates such as marital status, perceived stress, and social support^101^.

## Social factors specific to late life

Adults tend to maintain fewer peripheral social partners as they age^102^. It is hypothesized that an increasing awareness of time as limited influences older adults to prefer smaller and more emotionally satisfying social networks^103^. Despite changes in social network structure over time, the average degree of loneliness appears fairly constant from middle- to late-life^104^. In a sample of over 1600 older adults participating in the Health and Retirement Study, 43% reported feeling lonely^105^. Similarly to findings with younger and middle-ages adults, loneliness increases risk for depression in late life. For example, a study of elderly Finish adults found that loneliness predicted long-term trajectories of depression^106^. Additional social factors known to increase risk for LLD include bereavement, sleep disturbance, disability, prior depression, and female gender^107^. A meta-analysis of gender differences in LLD suggests that some of these effects are attenuated once sex differences in prevalence of widowhood, health, and socioeconomic status are accounted for^108^. Depression is also more common in older adults living in institutions compared to those living at home^109,110^.

## Cognitive factors affecting resilience in LLD

LLD is associated with risk of cognitive decline^111,112^, mild cognitive impairment (MCI)^113,114^, and dementia^111,115,116^. One possible explanation for this association is that LLD and cognitive decline are manifestations of the same underlying neuropathology. Indeed, both LLD and dementia are associated with reduced brain volume^117^, increased hippocampal atrophy^118^, increased white matter microstructural changes^119^, and altered structural and functional connectivity^63^. Research suggests that chronic stress-associated stimulation of the hypothalamic pituitary adrenal (HPA) axis and associated over-secretion of the stress hormone cortisol contribute to neurodegeneration^120,121^ that may increase risk for both LLD and cognitive decline. In addition, depressive symptoms may contribute to cognitive decline. Evidence supporting this hypothesis comes from a longitudinal study of 1764 older adults without cognitive impairment at baseline^122^. In that study, depressive symptoms predicted cognitive decline independent of the neuropathologic hallmarks of dementia. Other research indicates that psychological resilience may be neuroprotective. A recent study found significant associations between self-reported resilience and language performance among 288 adults with LLD^123^. In addition, the resilience factor grit was associated with greater structural integrity of the genu of the corpus callosum and cingulum, pathways implicated in cognitive and emotion regulation (*N* = 70)^124^.

The term *cognitive reserve* has been used to explain differences in susceptibility to cognitive decline resulting from brain aging, pathology, or insult^125^. Individual differences in cognitive reserve are determined by such factors as early-life general cognitive ability/intelligence, education level, occupation complexity, physical exercise, social engagement, and ongoing cognitive engagement^126^. These environmental and social factors are believed to enhance neural networks that promote neuroplasticity. Cognitive reserve may also serve as a psychological resilience factor. A recent systematic review representing data from over 37,000 older adults found that cognitive reserve decreased the association between cognitive impairment and depressed mood^127^. Of course, factors associated with cognitive reserve such as education are also associated with engagement in health-promoting behaviors that may further protect the individual against both depression and cognitive decline^128^.

Other research has attempted to identify cognitive factors that predict treatment response in LLD. One recent study found that impairment in response inhibition (a fundamental executive function) predicted poor antidepressant response in LLD^129^. Other studies have similarly found that baseline impairments in episodic working memory, processing speed, executive function, as well as severity of baseline white matter hyperintensities (WMH) predict decreased LLD improvement with antidepressant treatment^130^.

## Psychobiological resilience factors

### The stress response and LLD

Recent research has begun to investigate the biological mechanisms by which chronic stress increases risk for depression^131–135^. In a psychobiological framework, resilience can be defined as the adaptive maintenance of homeostasis in the face of stress or adversity^136^. Building psychobiological resilience begins with prenatal and early-life development^134^. Experimental studies in animals^136^ as well as observational studies in humans^137,138^ point to an inverted U-shape between early life challenges and adult stress reactivity, with moderate challenges in early life predicting optimal mental health in adulthood. Animal studies indicate that this so-called “early-life stress inoculation” decreases subsequent cortisol secretion and increases subsequent exploration of novel situations, cognitive control, and ventromedial prefrontal cortical volumes^138^. It is hypothesized that prefrontal myelination and cortical expansion induced by successful early-life coping lead to enduring adaptive cognitive and emotional changes^138^. By contrast, high levels of early-life adversity adversely impact attachment, personality, core beliefs, and coping tendencies, ultimately leading to enduring changes in endocrine, autonomic, and immunological processes^139^, and increasing vulnerability to depression^132,140^. Genetic factors also contribute. One study found that a functional polymorphism in the promoter region of the serotonin transporter (5-HTT) gene moderated the influence of both childhood stress and later stressful life events on risk for depression^141^.

One prominent hypothesis for how chronic stress increases risk for depression is through sustained activation and ultimate dysregulation of the HPA axis^142^. Of the stress hormones, cortisol has received the most research attention due to its widespread regulatory influence^143^. Research suggests that although uncontrollable stress initially amplifies cortisol secretion, sustained elevated levels of cortisol eventually suppress output of corticotropin-releasing hormone (CRH) and adrenocorticotropin hormone^143^, resulting in below-normal cortisol levels in chronically stressed populations^144^. This ultimate blunting of HPA axis responsivity is proposed to underlie the withdrawal and disengagement behaviors that often accompany chronic uncontrollable stress^144–146^. Disrupted HPA axis activity as evidenced by failure to suppress cortisol in the dexamethasone test predicts increased suicide risk in both MDD^147^ and LLD^148^, and recent research indicates that low mineralocorticoid receptor availability also increases risk for depression^149^.

In addition to altering cortisol secretion, sustained activation of the HPA axis results in deficient monoamine transmission, disruption of neurotrophic processes (e.g., the neuroprotective brain-derived neurotrophic factor (BDNF)), oxidative stress, widespread inflammatory processes, and neurodegeneration^136,142^. Dysfunction in serotonergic and dopaminergic transmission contribute to the common mood and cognitive symptoms observed in depression^142,150^. Once in motion, this stress-related biological cascade can be exacerbated by environmental factors (e.g., social isolation) or maladaptive coping behaviors (sedentary lifestyle, substance abuse)^134^.

### Neuropeptides

Multiple neuropeptides are known to modulate emotional processing. Neuropeptide Y (NPY) has been proposed as an endogenous mediator of resilience to stress-related psychiatric illness, including depression^151^. NPY plays a key role in the maintenance of homeostasis and has been implicated in diverse motivational, perceptual, and affective processes including circadian rhythm, anxiety, appetite, alcohol consumption, and pain perception^152^. NPY receptors are densely expressed in brain regions relevant to mood disorders including the cortex, hippocampus, and amygdala. Low NPY levels have been reported in MDD compared to healthy controls^153^, and genetic variation associated with low NPY expression increases risk for MDD^153,154^. Results of one study suggest that this effect may be mediated by increased neuronal response to affective stimuli in the medial prefrontal and anterior cingulate cortices among individuals with low-expression NPY genotypes^154^.

### Endocrine changes in aging and depression

One potential vulnerability for depression specific to older adults is age-associated decline in reproductive hormones. Perimenopause increases risk for both recurrent and new-onset depression^155^. Loss of normal estradiol (the primary circulating estrogen) cycling is proposed to account for this increased vulnerability via effects on neurotransmitter and mood regulatory systems^155^. Estrogen receptor polymorphisms have been associated with heightened depression risk in older women^156,157^, and maintenance of normal estrogen levels is important for several brain regions vulnerable to age-related changes^158^ (e.g., the prefrontal cortex (PFC) and hippocampus)^134,159,160^. Similarly, age-related reduction in dehydroepiandrosterone (DHEA) has been linked to depression, cognitive decline, reduced immune function, and decreased physical health^161,162^. Individuals with LLD demonstrate lower DHEA levels compared to non-depressed older adults, and DHEA levels increase with remission^163^.

### Cardiovascular markers

There is strong evidence for a bi-directional association between depression and cardiovascular disease. Prospective studies indicate that individuals with depression are at nearly twice the risk of developing cardiovascular disease and have nearly a three times higher mortality rate following a cardiac event^164,165^. Conversely, cardiovascular disease prospectively predicts depression^166,167^. Although unhealthy behaviors (e.g., unhealthy diet, lack of physical activity) undoubtedly contribute to this effect, lifestyle factors do not fully explain the relation between heart health and depression^168^.

Autonomic nervous system (ANS) dysregulation is one biological mechanism that may explain the link between cardiovascular risk and depression^169^. Heart rate variability (HRV) is a surrogate index of resting cardiac vagal outflow that represents the ability of the ANS to adapt to a changing psychological, social and physical environment^170^. Higher HRV is thought to reflect greater self-regulatory capacity (i.e., regulation of behavioral, cognitive, and emotional processes), and meta-analytic data suggest that this effect is larger for older compared to younger adults^171^. Recent research suggests that high HRV may serve as a biomarker of resilience to the development of stress-related disorders^172^ including depression^168,173^. However, age-related differences have been reported in the frequency of the HRV most predictive of depression. Whereas low high-frequency (HF)-HRV (reflecting parasympathetic activity^174^) is associated with depression among children^173^, adolescents^173^, and young adults^175^, only low low-frequency (LF)-HRV (reflecting both sympathetic and parasympathetic activity) appears associated with depression among older adults^168^. Decreased parasympathetic activity with age appears to result in decreased HRV in the general (primarily non-depressed) older adult population^176,177^, which may partially account for this finding.

### Inflammation in aging and depression

Cumulating evidence indicates immune and metabolic dysregulation among individuals with depression^178^. Immunometabolic dysregulation is associated with more severe and chronic depressive symptoms^179–181^ as well as decreased response to antidepressant treatment^182–184^, and may explain the increased prevalence of cardiovascular disease and diabetes in MDD^185^. While studies of younger adults typically show upregulation of metabolic processes in depression, studies of LLD report both upregulation and downregulation of these processes^186–189^. Both younger^190^ and older^191^ adults with depression show increased levels of inflammatory cytokines, secreted proteins that interact with virtually every depression-relevant neurophysiological domain (e.g., neurotransmitter metabolism, neuroendocrine function, and neural plasticity). Additional evidence for the role of inflammation in depression comes from studies indicating that (1) pro-inflammatory factors precede depressive symptom onset^191^, (2) antidepressant treatments reduce pro-inflammatory factors^192^, and (3) anti-cytokine therapy decreases depressive symptoms in placebo-controlled trials^193^. Experimental data indicate that acute psychosocial stress (e.g., public speaking, mental arithmetic) stimulates inflammatory signaling molecules^194^, and these responses are exaggerated in patients with depression^190^. Both childhood maltreatment^195^ and chronic stress in adulthood^196–198^ are associated with increased inflammation. Thus, inflammation may be one pathway by which these psychosocial factors increase risk for depression.

### Genetic factors in aging and depression

At least three genes have been associated with increased risk for LLD: the methionine (Met) allele of the neurotrophic factor BDNF^199^, APOE-e4 (involved in myelin repair and Aβ metabolism), and SLC6A4 (the short allele of the serotonin transporter 5-HTTLPR)^200^. BDNF is a protein that stimulates neurogenesis and is important for long-term memory. The Met variation of the BDNF gene is associated with decreased BDNF secretion^201^, poorer memory performance^202^, and increased risk for a range of neuropsychiatric disorders^203^. Interestingly, a meta-analysis found that the Met allele predicted MDD among men but not women^204^. Another meta-analysis found that the Met allele significantly moderated the effect of stressful life events on MDD risk, suggesting that Met carriers are more genetically sensitive to adverse life experiences^205^. A recent longitudinal study conducted with over 1000 older adults found that epigenetic regulation of the BDNF gene was associated with depression^206^. Another study of individuals with LLD found that the Met allele predicted poorer response to paroxetine, and that this effect was moderated by the cyclic AMP responsive element binding protein 1 (CREB1)^207^.

### Neuroimaging biomarkers of aging and depression

Emerging research evidence suggests that MDD is associated with reduced structural and functional plasticity^208,209^. Brain structures important for learning and applying adaptive coping strategies (e.g., the hippocampus and PFC) show atrophy in MDD^210–214^, possibly resulting from depression-related hypercortisolemia^215,216^. Similar abnormalities (i.e., decreased limbic structure volumes and reduced PFC activity) have been found in LLD^215,217–219^. However, research suggests that at least with regard to decreased hippocampal volumes^220^, these effects may be more pronounced for individuals with earlier depression onset. Narayan and colleagues propose that prior depressive episodes, aging, stress, hypercortisolemia, and reduced BDNF levels cause focal atrophy and may decrease the threshold for mood disorders in late life^221^.

### Neuroimaging biomarkers of emotion regulation, coping, and grit

Substantial neuroimaging research has investigated the neural networks implicated in emotion regulation and coping with stress. Despite the neural structures hypothesized to underlie emotion regulation being vulnerable to age-related decline^222^, behavioral evidence suggests that older adults have better emotion regulation capacity compared to younger adults^223^. Functional magnetic neuroimaging (fMRI) studies have documented increased activation of the PFC and amygdala^224,225^ in older vs. younger adults during tasks requiring emotion regulation tasks, possibly reflecting compensation for less efficient cognitive processing^224^.

Other studies have attempted to identify the neural correlates of adaptive coping with stress. For example, a study of 102 heathy adults found that the functional connectivity of regions associated with the default mode and anterior salience networks was associated with propensity to adopt various coping strategies (e.g., problem-focused, avoidant, social support seeking)^226^. Others have investigated individual differences in brain structure that relate to personality characteristics associated with resilience to depression. As reported above, grit has been associated with greater structural integrity of the genu of the corpus callosum and cingulum in LLD—pathways implicated in cognitive and emotion regulation^36,124^. Finally, several studies have documented neuroimaging correlates of self-reported resilience. Among healthy adults, self-reported resilience was correlated with decreased electroencephalogram (EEG) responsivity to adverse images^227^. In LLD, correlates included low amygdala blood perfusion at rest and greater functional connectivity between the amygdala and the ventral default mode network^228^.

### Structural brain changes and cerebrovascular disease in aging and depression

WMH are another related biological mechanism that may explain the link between cardio- and cerebrovascular disease and depression in late life. LLD is consistently associated with greater WMH severity^229,230^, and individuals with late onset exhibit greater WMH severity^220,231–234^ and greater cognitive impairment^234–236^ compared to those with first onset earlier in life. Depressed older adults who present with WMH are said to have “vascular depression”^237^, a subtype of depression characterized by cognitive deficits, psychomotor retardation, lack of insight, and disability disproportional to depression severity^238^. Such vascular abnormalities are linked to greater depressive symptom severity and poorer treatment response^239,240^. WMH are also strongly and independently associated with symptoms of apathy^241–245^.

### Psychobiological factors associated with early- vs. late-onset LLD

Several studies have investigated the clinical correlates of early- vs. late-onset LLD. One study investigated illness severity and symptoms, cognitive function, antecedent life events, physical health, genetic factors, and vascular health as a function of age of onset in 57 adults with LLD^246^. They found that early onset was associated with increased symptoms of anxiety and greater heritability. Several studies of LLD have found that early-onset recurrent illness predicts slower treatment responses and greater relapse compared to late onset^247^. This may be due to a greater number of depressive episodes, which is associated with the depletion of neural^248^, interpersonal^249,250^ and psychosocial resources^249,250^. These results are seemingly in contrast to the results of studies identifying characteristics associated with late onset (i.e., increased subcortical hyperintensities^234^, decreased cognitive performance^234^), which have also been associated with inadequate treatment response^239,240^.

## Resilience-enhancing interventions

Resilience-enhancing interventions can be implemented either preventatively to reduce susceptibility to MDD or as a treatment following MDD onset. Prevention strategies promote well-being even in the absence of current psychopathology, and can be applied both to healthy individuals or to those at high risk (i.e., those with chronic stress, trauma, or history of prior depressive episodes). The field of positive psychology^251^ defines well-being as not the absence of a mental disorder, but rather the presence of well-being, and advocates for the widespread application of such techniques regardless of the presence or absence of psychopathology.

### Positive psychology interventions

Positive psychology interventions (PPIs) are “treatment methods or intentional activities that aim to cultivate positive feelings, behaviors, or cognitions”^252^(p. 468). PPIs typically target hedonic well-being (e.g., positive affect, life satisfaction, happiness)^253^, eudaimonic well-being (e.g., self-acceptance, positive relations, autonomy, purpose in life)^254,255^, or both, and are typically amenable to self-administration. Such approaches vary widely in intensity, ranging from a several minute-long daily gratitude journal to more in-depth journal exercises, meditations, and intentional social behaviors. Research suggests that PPIs not only increase well-being, but also improve the individual’s capacity to “bounce back” from adversity. A recent meta-analysis of PPIs including data from over four thousand adults indicated a highly significant, moderate effect of PPIs on both well-being (*r* = 0.29) and depressive symptoms (*r* = 0.31)^252^. PPIs included expressing gratitude^256,257^, reflecting upon one’s ideal future self^257–260^, identifying one’s strengths^256^, practicing mindfulness^261^, and practicing compassion/ acts of kindness^262–265^. Interventions delivered individually were most effective, followed by those administered in a group, then by self-administered PPIs. The efficacy of PPIs increased linearly with participant age, and depressed individuals benefitted more than non-depressed individuals^252^. Another PPI shown to improve mood in individuals at risk for depression due to trauma exposure is “benefit finding”, or the intentional identification of positive ways in which their lives have changed as a result of a traumatic event^266,267^. Bower and colleagues^268^ propose an integrative conceptual model in which benefit finding promotes the development of interpersonal resources (e.g., adaptive coping strategies including cognitive re-appraisal; increased self-efficacy for coping with stress; more salient sense of one’s values/purpose) and intrapersonal resources (e.g., enhancement of social relationships) that facilitate more adaptive responses to future stressors. More broadly, PPIs that increase one’s feeling of connection to others (e.g., expressive gratitude, practicing compassion may not only directly improve mood but also exert beneficial neuroendocrine, cardiovascular, and immune systems changes^269^. PPIs aimed at enhancing meaning/purpose (e.g., reflecting upon one’s ideal future self; identifying strengths) are also thought to improve markers of immune functioning^270^. In addition, it is possible that the positive psychological changes elicited through PPIs have their own mechanistic pathways distinct from those associated with distress—e.g., parasympathetic nervous system activity, growth factors, and other neuroendocrine factors such as oxytocin^271^. Although PPIs are not recommended as a stand-alone treatment for moderate-to-severe MDD, research suggests that PPIs may be useful for the prevention or adjunct treatment of depression^252^.

### Meditation

Meditation refers to a category of mind–body techniques most commonly involving the directed focus of attention. One form of Buddhist meditation techniques that has been adapted to a variety of secular settings in the West is *mindfulness*—intentional, non-judgmental, present-focused awareness^272^. A recent meta-analysis indicated that mindfulness-based interventions are significantly more effective than psychoeducation, supportive psychotherapy, relaxation training, and guided imagery for improving a range of mental and physical health symptoms, with the largest effects demonstrated for mental health symptoms including depression^273^. A recent review of randomized controlled trials (RCTs) of mindfulness-based interventions for older adults concluded that Mindfulness-Based Stress Reduction (MBSR) is effective for improving symptoms of depression, anxiety, positive affect, insomnia, chronic pain, memory, and executive functioning in late life^274^. Other research conducted with adults with MDD has found that MBSR is effective for preventing relapse in those with a history of three or more depressive episodes^275^. A recent systematic review evaluating the biopsychological mechanisms by which mindfulness practice affects clinical outcomes concluded that decreases in cognitive reactivity, emotional reactivity, rumination, and worry may mediate the effect of mindfulness interventions on mental health^276^. Mindfulness practice appears to alter both brain structure and function, most notably in areas related to attentional control, self-awareness, and emotion regulation^277–279^. In addition, mindfulness interventions may protect against age-related decreases in gray matter volume^280^, attention performance^281^, and cellular aging^282^.

Other forms of yogic-style meditation include those involving repetition of sounds (mantras) or hand positions (mudras). A recent study found that Kirtan Kriya (which combines mantras and mudras) was more effective for improving mental health and cognitive functioning in dementia caregivers compared to passive listening to relaxing music^283^. Further, Kirtan Kriya reversed the pattern of increased pro-inflammatory cytokine and decreased innate antiviral response gene transcription observed in chronically stressed individuals. Results of this study indicate inflammatory and antiviral transcription pathways as one mechanism by which meditation may increase resilience in older adults^284^. Another randomized study of mantra meditation for elderly women with hypertension found that chanting significantly reduced depression, anxiety, stress, blood pressure, and cognitive impairment^285^. Studies investigating the neurohemodynamic correlates of mantra meditation suggest that deactivation of the limbic system may account for observed decreases in physiological arousal and improvements in well-being^286^.

### Psychotherapy

Meta-analytic data suggest that psychotherapy is similarly effective as pharmacological approaches in the treatment of LLD^287,288^, with a number needed to treat of 3 (ref. ^288^). A meta-analysis of RCTs comparing psychotherapy for LLD to various control conditions found the largest effects for cognitive behavior therapy (CBT; *g* = 0.45), problem-solving therapy (*g* = 0.46), and life review therapy (*g* = 0.59)^287^. A more recent review focused on mindfulness-based CBT found such interventions to be similarly effective for improving late-life depressive symptoms (*g* = 0.55)^289^. Results of another meta-analysis suggest that music therapy significantly augments the efficacy of standard treatments for LLD^290^. Because alterations of the HPA axis and the ANS appear to be involved in the development and maintenance of depression, it is conceivable that these dysregulations may interact with psychotherapeutic treatment to influence outcomes. A meta-analysis that attempted to investigate the effect of psychotherapy on HPA and ANS regulation in adults with mixed mental health disorders concluded that this effect could not be estimated due to the large degree of heterogeneity of methodologies across studies^291^.

### Movement-based interventions

Physical exercise is effective for the treatment of MDD, with effect sizes similar to those observed with pharmacological and psychological therapies^292^. A recent systematic review confirmed that physical activity interventions are also effective in LLD^293^. In addition to the documented effects on mood, physical activity is associated with improved balance, greater strength, and decreased disability. One mechanism by which exercise interventions affect both physical and mental health may be via reduction in inflammatory cytokine responses resulting from hemodynamic hormonal changes during physical activity^294^. Movement-based interventions that also incorporate mindfulness are referred to as mind–body therapies (MBTs). A recent review of the efficacy of such interventions for late-life mood and cognitive disorders concluded that MBTs such as yoga and tai chi may outperform conventional physical exercise with regard to effects on mood, QOL, and cognitive functioning^295^. A review of the neural mechanisms of movement-based vs. stationary meditation found that these two approaches affect multiple common brain regions including those involved in attention, memory, awareness, and emotional processing^296^. Yoga appears to reduce HPA axis activity in younger healthy adults^297,298^ as well as in sedentary community-dwelling older adults without depression^299^. It is possible that restoration of HPA axis dysregulation may be one mechanism by which MBTs improve mood in older adults with depressive symptoms^283,300–302^.

### Pharmacotherapy

Second-generation antidepressant medications, including selective serotonin reuptake inhibitors (SSRIs), bupropion, mirtazapine, venlafaxine, and duloxetine are the most commonly prescribed pharmacological treatments for LLD. A recent meta-analysis of second-generation antidepressants vs. placebo for treatment of LLD found that response rates were modest (44% for antidepressant vs. 35% for placebo)^3^. Response rates were higher for 10–12-week trials (55%) compared to 6–8-week trials (38%)^3^. However, results of another meta-analysis comparing placebo-controlled vs. comparator trials in LLD suggest that these effects may underestimate those in clinical settings, when patient expectations of improvement may be higher^303^. Discontinuation rates due to adverse events associated with second-generation antidepressant medication range from 8–27%, compared to 1–11% for placebo^3^. The precise neurophysiological mechanisms of antidepressant medications remain unknown. However, meta-analytic data indicate normalization of neural responses to positive and negative stimuli in limbic regions as well as increased self-regulatory potential via changes in the dorsolateral PFC^304^.

### Electroconvulsive therapy

Electroconvulsive therapy (ECT) is widely used for the management of severe and refractory MDD^305^. As the most effective biological treatment for major depression, ECT is associated with remission rates of 51% (ref. ^306^) and standardized effect sizes of 0.91 compared to sham ECT^307^. Reviews indicate that ECT is especially effective in older adult patients, with remission rates reaching 73–90% (refs. ^308–312^). In addition, psychotic and melancholic features predict greater response^312^. Use of ECT for LLD is safe, well-tolerated, and effective for improving cognition and psychomotor symptoms^313^. Maintenance ECT, with treatments spaced over weekly to monthly intervals, is often used for relapse prevention^314^. Recommendations for optimal administration of ECT for LLD can be found elsewhere^312^. The results of one study investigating the neurological effects of ECT suggest that increases in frontal white matter fractional anisotropy (FA) (typically reduced in LLD compared to age-matched controls^315^) may account for ECT’s antidepressant effect^315^. Another study found gray matter volume increases in the right caudate nucleus with ECT treatment for LLD, a change which correlated with improved psychomotor function^313^.

### HRV biofeedback

Small uncontrolled studies have shown some promise for HRV biofeedback in the treatment of MDD^316,317^, and one RCT found that HRV biofeedback significantly improved depressive symptoms in first-time cardiac surgery patients compared to usual care^318^. It is thought that strengthened homeostasis or effects on the vagal afferent pathway to frontal cortical areas may account for this effect^319^. However, no RCTs to date have examined the efficacy of HRV biofeedback in LLD.

## Directions for future research and public policy

During this critical time of accelerated global aging, understanding factors that promote resilience vs. risk for the development of LLD and related comorbidities is more important than ever. Continued research in this area is essential for informing practice and public policy to promote successful aging, reduce disability, and mitigate rising healthcare costs. One fruitful area for future research is the investigation of individual characteristics that moderate risk factors for LLD. For example, research suggests that risk factors for depression may vary according to generation^110^, gender, and cultural origin. Variables such as self-esteem and self-efficacy are highly culturally and contextually dependent, such that the efficacy of interventions targeting these processes will likely vary across cultures. Indeed, PPIs as a whole appear more effective among members of individualist cultures^320^, perhaps because the rationale for such approaches resonates more strongly with individuals from cultures endorsing the pursuit of individual happiness. It is possible that individuals from collectivist cultures may derive greater benefit from prosocial and other-focused PPIs (e.g., performing acts of kindness, writing a letter of gratitude), compared to self-focused PPIs (e.g., reflecting on personal strengths)^252^.

Similarly, continued investigation of the biological factors that increase or mitigate risk for depression is of paramount importance. Results of a recent meta-analysis suggest that depressed individuals with higher baseline cortisol levels are less responsive to psychological therapy^321^. Because elevated cortisol has been linked to concentration and memory difficulties^322^, the authors propose that difficulty engaging in learning processes may reduce the efficacy of psychotherapy in this subset of MDD patients. Additional research is needed to identify other biomarkers of depression “subtypes” as well as determine as well as the interventions most effective for each. Continued work in this area is essential for the development of more effective approaches to the treatment of LLD.

Resilience-enhancing interventions can be applied not only at the individual level, but also at the level of the family, organization, or community. A recent systematic review of family-oriented interventions found that dyadic interventions such as ecosystems therapy, psychoeducation, family counseling, behavioral therapy, and CBT are feasible and effective for the treatment of LLD^323^. In addition, the results of two pilot studies suggest that mindfulness training delivered jointly to older adult patients and their caregivers is effective for reducing depressive symptoms^324–326^.

Other research has identified potentially useful directions for organizations that wish to promote resilience in their older adult volunteers. A recent study of almost 400 older adult volunteers identified adequate training, ongoing support, and greater volunteer choice as predictors of larger volunteer mental health benefits^327^. This effect was mediated by the volunteer’s belief that their work had meaningfully contributed to the well-being of others^327^.

Factors influencing risk for LLD can also be identified at the community and public policy levels. Because loneliness affects nearly half of the Americans^99^ and independently predicts severity and course of LLD^328^, loneliness is a promising target for intervention. Of note, a recent review of seven RCTs found that social robot interventions may be effective for helping to alleviate depressive symptoms in older adults when used during group activities^329^. Programs to promote greater integration of older adults into their communities (e.g., by facilitating the sharing of meals, joint physical activity, support groups, or interactive volunteer work) are recommended. Of course, factors such as a balanced diet, physical activity, and sufficient sleep are also of paramount importance. As such, public policies that provide food stamps and low-cost healthy food options, safe opportunities for physical exercise, preventative medical care, and financial assistance to those older adults in need will reduce the economic, social, and individual QOL burden of LLD.

## Conclusion

With the global population rapidly aging and depression as the leading cause of disability worldwide, identification of factors that increase resilience to LLD is of paramount public health importance. Risk for LLD begins as early as embryonic development and is determined by complex interactions between biological and psychological factors. Research has elucidated both trait-like psychological factors that identify those at greatest risk and psychosocial processes that may be fruitful targets for intervention. Psychobiological factors include endocrine, genetic, inflammatory, neural, and cardiovascular processes that bidirectionally interact to affect LLD risk. Intervention research suggests that MBTs (including yoga^299,330,331^, MBSR^332^, tai chi^333,334^, qigong^302^, and meditation^335,336^) have potential for improving HPA axis regulation^299^ and depressive symptoms in older adults with depression^283,300–302^ as well as in non-depressed older adults^330–332,334–336^.

Additional research into the biophysiological mechanisms by which psychosocial processes affect risk for LLD will identify novel targets for intervention. In addition, continued research testing the efficacy of interventions designed to enhance resilience to LLD is critically important. Further work in this area has potential to greatly increase QOL, reduce morbidity, and decrease healthcare costs for aging adults.
